# GEOGRAPHIC AND DEMOGRAPHIC PREDICTORS OF SELF-RATED HEALTH IN OLDER RURAL AFRICAN AMERICANS

**DOI:** 10.1093/geroni/igad104.0431

**Published:** 2023-12-21

**Authors:** Darby Mackenstadt, Carolyn Adams-Price, Sarah Israel

**Affiliations:** Mississippi State University, Mississippi State, Mississippi, United States; Mississippi State University, Mississippi State, Mississippi, United States; Mississippi State University, Starkville, Mississippi, United States

## Abstract

Person-Environment (P-E) fit is a major tenet of environmental gerontology and can predict aspects of physical and mental health. Rural older adults may vary greatly in their P-E fit, but their in-group differences have yet to be explored. The current study examines predictors of self-rated health in middle and older adult African Americans in rural Mississippi. Predictors of self-rated health examined included distance from grocery store, age, education, and income. Participants were 47 African Americans (20 males) over 50 (M = 65.2) who were recruited from two rural, adjacent Mississippi Counties and lived at least five miles outside of a micropolitan town. Consistent with literature on health disparities in African American communities, participants on average rated their health as average to low. Results from a multiple regression model indicate that education and distance to grocery stores are significant predictors of subjective health, where more education and being further from a grocery store predicted higher self-rated health. It appears that within this sample, the further from a grocery store they were, the better the P-E fit. This may be due to an increase in gardening or farming, increased necessity for physical activity, or other aspects of this environment that promote healthy behaviors, despite being further from resources. Future research should continue to pursue aspects that add to P-E fit in rural older adults.

